# Molecular Docking Suggests the Targets of Anti-Mycobacterial Natural Products

**DOI:** 10.3390/molecules26020475

**Published:** 2021-01-18

**Authors:** Rafael Baptista, Sumana Bhowmick, Jianying Shen, Luis A. J. Mur

**Affiliations:** 1 Institute of Biological, Environmental and Rural Sciences, Penglais Campus, Aberystwyth University, Aberystwyth, Wales SY23 2DA, UK; rafaelb4@gmail.com (R.B.); sub23@aber.ac.uk (S.B.); 2 Artemisinin Research Center, Institute of Chinese Materia Medica, China Academy of Chinese Medical Sciences, Beijing 100700, China

**Keywords:** tuberculosis, reverse docking, natural products, anti-mycobacterial agents

## Abstract

Tuberculosis (TB) is a major global threat, mostly due to the development of antibiotic-resistant forms of *Mycobacterium tuberculosis,* the causal agent of the disease. Driven by the pressing need for new anti-mycobacterial agents several natural products (NPs) have been shown to have in vitro activities against *M. tuberculosis*. The utility of any NP as a drug lead is augmented when the anti-mycobacterial target(s) is unknown. To suggest these, we used a molecular reverse docking approach to predict the interactions of 53 selected anti-mycobacterial NPs against known “druggable” mycobacterial targets ClpP1P2, DprE1, InhA, KasA, PanK, PknB and Pks13. The docking scores/binding free energies were predicted and calculated using AutoDock Vina along with physicochemical and structural properties of the NPs, using PaDEL descriptors. These were compared to the established inhibitor (control) drugs for each mycobacterial target. The specific interactions of the bisbenzylisoquinoline alkaloids 2-nortiliacorinine, tiliacorine and 13′-bromotiliacorinine against the targets PknB and DprE1 (−11.4, −10.9 and −9.8 kcal·mol^−1^; −12.7, −10.9 and −10.3 kcal·mol^−1^, respectively) and the lignan *α*-cubebin and Pks13 (−11.0 kcal·mol^−1^) had significantly superior docking scores compared to controls. Our approach can be used to suggest predicted targets for the NP to be validated experimentally, but these in silico steps are likely to facilitate drug optimization.

## 1. Introduction

Tuberculosis (TB) is the leading cause of death from infectious diseases, with 10 million new cases in 2017. About 1.7 billion people are estimated to have latent TB infection, and are at risk of developing active TB disease during their lifetime [1]. The emergence of multidrug-resistant (MDR) and extremely drug-resistant (XDR) TB is primarily due to the improper use of the first-line anti-tubercular drug. The increased prevalence of such strains has become a major obstacle in the treatment of TB and also a serious financial burden on the health care sector. As a result, there is an urgent need for new cost-effective anti-TB drugs with new mechanisms of action and less chance of developing resistance [2].

TB drug discovery has been based on the use of combinatorial chemistry and high-throughput screening strategies in drug discovery, but recently, there has been an increased interest in plant-based natural products (NP) as drugs [2]. Plants are an important source of secondary metabolites that can have enormous therapeutic potential. They are still used in traditional medicine in such nations as China and in economically developing countries. Often knowledge of medicinal plants is passed verbally from generation-to-generation without any proper documentation or scientific validation. However, medicinal plants still represent a resource that can be further explored for potential “hit” compounds with significant biological activity, i.e., drug leads [3]. These hit compounds are typically found in biochemically complex extracts, and their identification can be considered to be equivalent to searching for a “needle in a haystack”. This is usually approached through sequential rounds of bioassay informed purification but could be considerably accelerated if candidate chemicals could be screened against known and “druggable” drug targets. Crucially, the identification of these targets facilitates drug optimization for improved efficacy and such reduced cytotoxicity [2].

Reverse docking is widely used to model interactions at the atomic level between a small molecule (ligand) and a known macromolecule [4]. Molecular docking and other bioinformatic tools represent cost-effective approaches to screen potential compounds prior to in vitro cell culture-based assays or chemical modifications to accelerate the overall drug discovery process. The importance of the reverse docking technique was evident in its recent application in the identification of p38-alpha kinase as the molecular target of anti-inflammatory natural products hybrids. This approach was directly linked to the identification of esculentoside A’s target and mode of action [5,6]. In this present study, we exploited the existing knowledge of anti-tubercular drug targets to predict the potential modes of action of NPs known to have activity against TB. Seven molecular targets of *M. tuberculosis*—ClpP1P2, DprE1, InhA, KasA, PanK, PknB and Pks13—were selected as these are essential for bacterial survival, and their inhibition will affect mycobacterial metabolism [7,8,9,10,11,12,13,14,15,16,17,18]. We herein predict the binding of the NPs in comparison with the established inhibitor of the molecular target, which was referred to as the control. We show that the specific interactions of the bisbenzylisoquinoline alkaloids 2-nortiliacorinine, tiliacorine and 13’-bromotiliacorinine against PknB and DprE1 and the lignan *α*-cubebin with Pks13 had significantly superior docking scores. The predicted interactions here reported should facilitate in vitro validation studies.

## 2. Results

**Figure 1:**
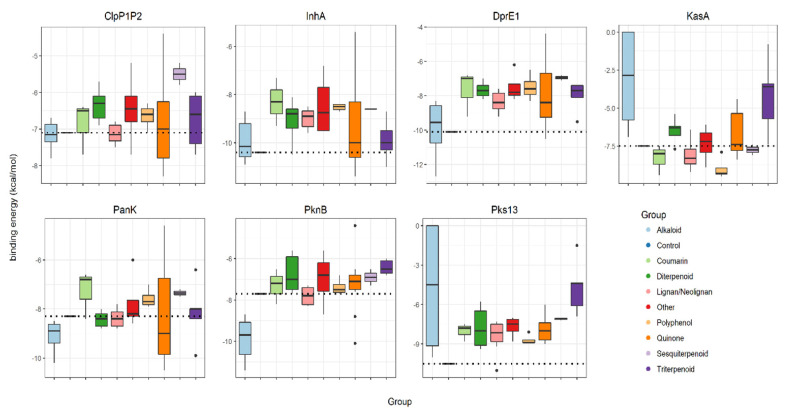
Binding energies (kcal·mol^−1^) of groups of selected natural products (alkaloids, coumarins, diterpenoids, lignans/neolignans, polyphenols, quinones, sesquiterpenoids, triterpenoids and others) and controls (represented with dashed lines) against mycobacterial targets ClpP1P2, InhA, DprE1, KasA, PanK, PknB and Pks13. Control inhibitors of each protein are, respectively, ZIL (N-[(benzyloxy)carbonyl]-l-isoleucyl-l-leucine), BTZ043 (bedaquiline), isoniazid, TLM (thiolactomycin), ZVT(2-chloro-*N*-[1-(5-{[2-(4-fluorophenoxy)ethyl]sulfanyl}-4-methyl-4*H*-1,2,4-triazol-3-yl)ethyl]benzamide),MIX(1,4-dihydroxy-5,8-bis({2-[(2-hydroxyethyl)amino]ethyl}amino)-9,10-antracenedione)andI28(ethyl-5-hydroxy-4-[(4-methylpiperidin-1-yl)methyl]-2-phenyl-1-benzofuran-3-carboxylate).

A total of 53 NPs with reported anti-mycobacterial activity (≤100 mg·mL^−1^) were selected [3] (Table 1). These were subjected to a series of in silico predictions to assess their “druggability” and suggest their targets. The 53 NPs were organized into chemical classes and then assessed for their individual binding energy against established antimicrobial target proteins, ClpP1P2, DprE1, InhA, KasA, PanK, PknB and Pks13, which were retrieved from Protein Data Bank (PDB). For ease of comparison, the binding energies associated with all groups of studied NPs against each mycobacterial target are given as box-plots and compared to the binding of the known anti-TB drug hit for each protein (Figure 1, control bindings are shown with a dashed line).

Three targets, InhA, Pks13 and DprE1, exhibited poor binding to all of the studied NPs. Only a few alkaloids and quinones exhibited lower energies (−11.4 to −10.5 kcal·mol^−1^) than the control drug isoniazid (−10.4 kcal·mol^−1^) against InhA. Considering Pks13, only one neolignan displayed lower energy (−11.0 kcal·mol^−1^) than the control drug I28 (ethyl 5-hydroxy-4-[(4-methylpiperidin-1-yl)methyl]-2-phenyl-1-benzofuran-3-carboxylate) (−10.5 kcal·mol^−1^). Similarly, only alkaloids displayed favorable binding energies (−12.7 to −10.2 kcal·mol^−1^), compared to the control, BTZ043 (bedaquiline) (−10.1 kcal·mol^−1^) against DprE1. Indeed, alkaloids exhibited very low binding energies (−11.4 to −8.7 kcal·mol^−1^) against PknB, when compared with the control inhibitor MIX (1, 4-dihydroxy-5,8-bis({2-[(2-hydroxyethyl)amino]ethyl}amino)-9,10-antracenedione) (−7.7 kcal·mol^−1^). In contrast, KasA and ClpP1P2 were shown to have some binding energy to a wide range of natural product classes. For KasA and ClpP1P2, binding was seen with coumarins, lignans/neolignans, polyphenols and quinones. KasA is also bound by sesquiterpenoids and ClpP1P2, by triterpenoids. Although the PanK is predicted to bind to different classes of NPs, the alkaloids and quinones had lower binding energies (−10.5 to −8.5 kcal·mol^−1^) compared with the control ZVT (2-chloro-*N*-[1-(5-{[2-(4-fluorophenoxy)ethyl] sulfanyl}-4-methyl-4*H*-1,2,4-triazol-3-yl)ethyl]benzamide) (−8.3 kcal·mol^−1^).

The average of MW presented by conventional anti-TB drugs [48] (358.5 g·mol^−1^) contrasted with our NPs that typically had lower binding energies (MW > 500 g·mol^−1^). However, the number of H-bonds acceptors of the NPs matched those of H-bonds acceptors of conventional anti-TB drugs [48], the majority of which were below 10 H-bonds acceptors. The same is observed with the number of rotational bonds, since 88% of conventional anti-TB drugs have less than 10 rotational bonds [48].

PaDEL-Descriptor was used to assess the key physicochemical properties necessary for an optimal binding between the NP with ClpP1P2, DprE1, InhA, KasA, PanK, PknB and Pks13, compared to each respective control drug. PaDEL-Descriptor provided molecular weight (MW), partition coefficient (xLogP), rotatable bonds (nRotB), H-bond donors (nHBDon_Lipinski), H-bond acceptors (nHBAcc_Lipinski) and topological polar surface area (TopoPSA) (Supplementary Table S3). All components were assessed and checked for toxicity and drug-likeness employing SWISS-ADME [49] including Lipinsky, Ghose, Veber, Egan, Muegge, PAINS [50], and Brenk filters [51]. SwissADME evaluation did not post PAINS alert for the molecules with higer binding energy (Supplementary Table S4).

For ClpP1P2, InhA and PanK, there was a clear tendency for molecules with a higher topological polar surface area to have more favorable binding energies. This is due, in part, to the low binding energies of quinones against these three protein targets (Supplementary Figure S1). Higher MW appeared to have lower binding energies against ClpP1P2, InhA, DprE1, PanK and PknB. In this higher MW category of natural product, lower binding energies, usually lower than the control inhibitor, were mostly seen with triterpenoids and sesquiterpenoids (Supplementary Figure S2). When the lipophilicities of the NPs were analyzed compared to binding energies, no particular tendency was observed (Supplementary Figure S3). The NP with more favorable binding energies did not exhibit distinctive partition coefficients as indicated by xLogP values. For PanK, DrpE1 and PknB, a higher number of H-bond acceptors (maximum of 8) was associated with lower binding energies. No similar trend was seen for the number of H-bond donors (Supplementary Figures S4 and S5). NPs with smaller rotation bonds were often linked to lower binding energies (Supplementary Figure S6).

**Figure 2:**
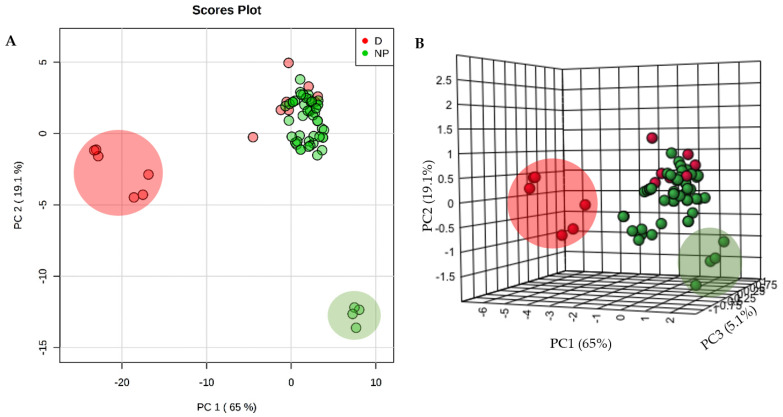
2D-PCA (**A**) and 3D-PCA (**B**) of the physicochemical parameters of 53 analyzed natural products (NP) and 14 anti-TB drugs (D). In one cluster, NP and D share similar physical and chemical properties, but two other clusters are unique of D (larger red circle) and another for NPs (larger green circle).

**Figure 3:**
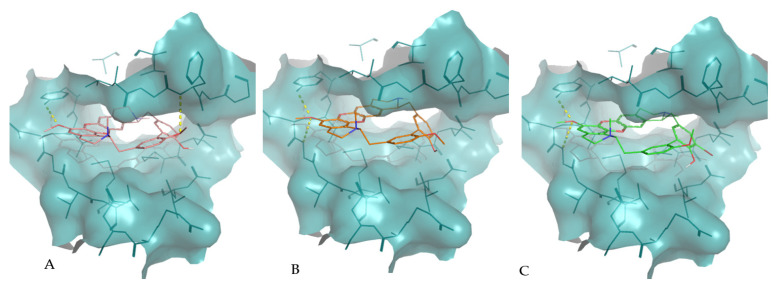
Molecular interactions of the best docking positions of tiliacorine (**A**), nortiliacorinine (**B**) and 13′-bromotiliacorine (**C**) against PknB. Hydrogen bonds are shown as yellow dashed lines.

Unsupervised principal component analysis (PCA) was used to provide a multivariate comparison of the physicochemical parameters of the selected NPs and 14 licensed anti-TB drugs (Figure 2). There was a large clustering of most NPs and anti-TB drugs, suggesting a significant commonality of properties. However, six anti-TB drugs (isoniazid, ethambutol, streptomycin, kanamycin, amikacin and levofloxacin, large red circle in Figure 3) do not cluster with the NPs mainly due to their high hydrophilicity. The three aminoglycosides—streptomycin, kanamycin and amikacin—also exhibit a high number of H-bonds donors (*n* > 10), which does not conform to one of “Lipinski’s rule of five”. Some NPs (selina-3,7(11)-diene, abietane and α-curcumene), represented in a large green circle in Figure 3, possessed distinctive chemical properties due to lack of any H-bond acceptors or donors. This would exclude them from being possible drug candidates without further derivatization.

Subsequently, a structural study was undertaken with the NPs that exhibited the most favorable anti-mycobacterial profiles, i.e., show lower energies than the control inhibitor (Figure 1). Thus, the interaction between the bisbenzylisoquinoline alkaloids 2-nortiliacorinine, tiliacorine and 13′-bromotiliacorinine against the targets PknB and DprE1 were modeled.

**Figure 4:**
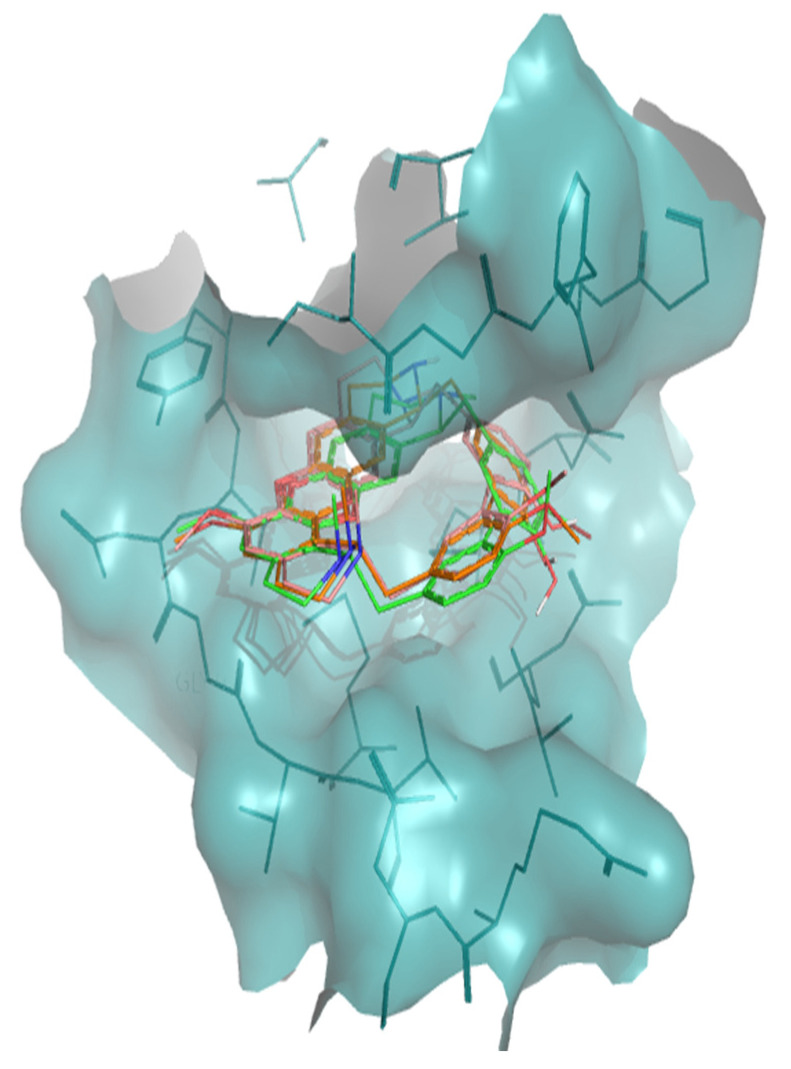
Superposition of the best docking positions of tiliacorine (pink), nortiliacorinine (orange) and 13′-bromotiliacorine (green) against PknB.

The interaction of tiliacorine, nortiliacorinine and 13′-bromotiliacorine with PknB is shown in Figure 3 and exhibited binding energies of −11.4, −10.9, and −9.8 kcal·mol^−1^, respectively. These values are significantly lower than the binding energy found for the control drug, MIX (−7.7 kcal·mol^−1^). The best docking positions of each of the three NPs were compared, and these showed considerable overlap (Figure 4). Such commonality of interaction could be related to inhibitory function and could guide drug optimization. In particular, a key feature here revealed is the interactions of the hydrophobic core of these NPs with PnkB 49 a feature also seen with the planar dihydroxyanthraquinone moiety of the control drug.

**Figure 5:**
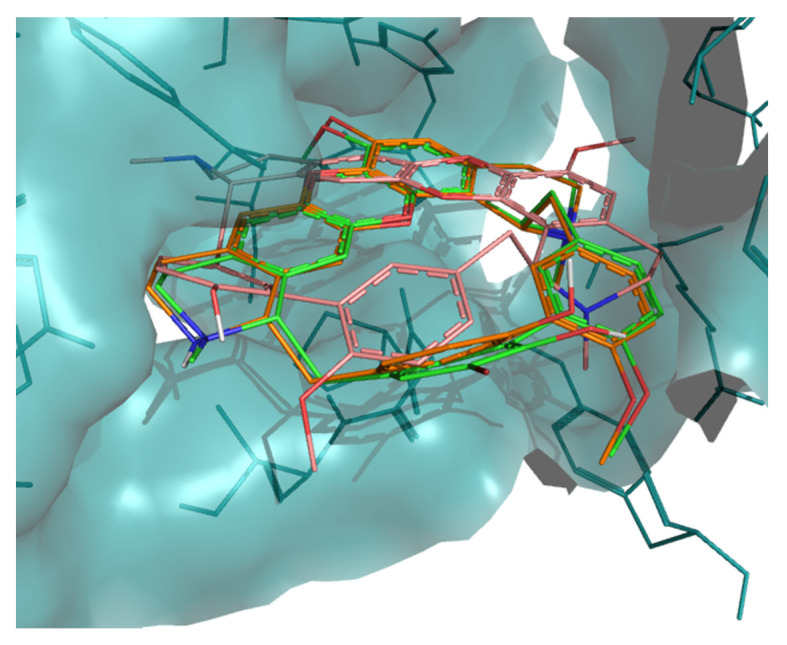
Superposition of best docking position of tiliacorine (pink), nortiliacorinine (orange) and 13′-bromotiliacorine (green) against DprE1.

The predicted interactions of tiliacorine, nortiliacorinine and 13′-bromotiliacorine with DprE1 were also visualized (Figure 5). Again, the interactions for all these NPs appeared to nearly superimpose. These showed better binding energies against DprE1, −12.7, −10.9, and −10.3 kcal·mol^−1^, respectively, than the benzothiazinethione drug control BTZ043 (−10.1 kcal·mol^−1^). The binding of DprE1 to tiliacorine, nortiliacorinine and 13′-bromotiliacorine, is stabilized by several non-covalent interactions. The LigPlot+ analysis shows that key van der Waals interactions with the residues Trp230, Val365, Lys367, Lys134, Tyr415, His132, Pro116, Ile131, Ala417, Lys418, Arg58, Thr118, Trp16, Tyr60, Gly117 and Tyr314 are responsible for the low binding energies of these structures with DprE1.

**Figure 6:**
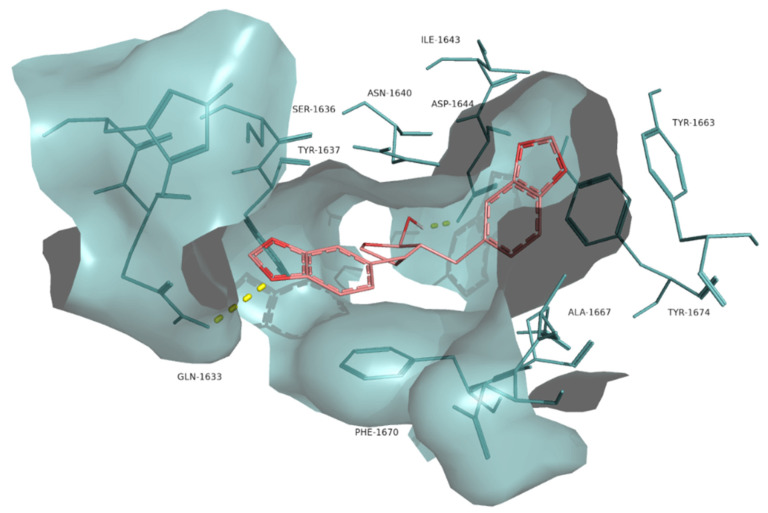
Molecular interactions of the best docking position of α-cubebin against Pks13. Hydrogen bonds are evidenced with yellow dashed lines.

The interaction between the lignan α-cubebin and Pks13 was examined (Figure 6) as it had a lower docking scoring (−11.0 kcal·mol^−1^) compared to the control I28 (−10.5 kcal·mol^−1^). α-cubebin interacts with Pks13 via two H-bonds with the residues Asp1644 and Gln1633 and several hydrophobic interactions with the residues Tyr1637, Ser1636, Phe1670, Ile1643, Tyr1663, Tyr1674, Ala1667, Asn1640 and Arg1641. The interaction with the residues Tyr1663, Tyr1674, Asn1640, Asp1644 and Gln1633 are also key features in the binding of the control drug I28 against Pks13 [10].

## 3. Discussion

Predictions of molecular docking are now well-established when assessing the interactions between ligands and targets. The use of docking approaches has been facilitated by the development of suitable software such as GOLD, FlexX, FRED, DOCK and particularly, AutoDock Vina [52,53]. Such in silico reverse docking provides a numerical estimate of the likelihood of interaction of a compound to its target. This approach can be extended to identify the proteins which are likely in vivo binding sites, and therefore possible modes of action [54,55,56]. For example, the target of the antibacterial and anti-fungal natural product scytoscalarol was found to dock with EmbC, and this was linked with anti-mycobacterial activity. Other compounds such as the *β*-carboline alkaloids 8-hydroxymanzamine A and manzamine A were found to bind to the oxidoreductase InhA.

We here demonstrate how reverse docking can be used to assess large numbers of anti-mycobacterial NPs to suggest key interactions and imply a mode of action. Our approach was to examine the literature for NPs with anti-mycobacterial activities, but whose targets had not been previously characterized. Then, proteins known to be targeted by established anti-mycobacterial drug leads were screened using the NP chemical structures. The aim was to identify natural product interactions whose docking energies were as good as, or superior to, the established drug lead. The “druggable” mycobacterial targets ClpP1P2, DprE1, InhA, KasA, PanK, PknB and Pks13, were all known to play important roles in maintaining mycobacterial viability. ClpP1P2 carries out the energy-dependent degradation of abnormal proteins within the cells during in vitro growth and infection [57]. DprE1 is a decaprenylphosphoryl-d-ribose oxidase involved in the biosynthesis of decaprenylphosphoryl-d-arabinose, an essential component of the mycobacterial cell wall and thus is essential for cell growth and survival [11,58]. InhA is a known target of isoniazid, a first-line anti-tuberculosis drug essential for the synthesis of mycolic acids. KasA is one of the enzymes responsible for the elongation of C16–26 fatty acyl primers in the FAS-II system for mycolic acid production of *M. tuberculosis* [59]. Pantothenate kinase (PanK) is a ubiquitous and essential enzyme that catalyzes the first step of the coenzyme A biosynthetic pathway [17]. PknB is a very well-characterized mycobacterial serine/threonine-protein kinase that determines cell shape, morphology and possibly cell division [9]. Pks13 is a polyketide synthase that catalyzes the final condensation step in mycolic acid biosynthesis and is therefore essential for mycobacterial growth [60].

A key aspect of our approach was to identify several “drug-like” properties of the NPs and compare them with conventional anti-TB drugs [21]. Our analyses first assessed the chemical space occupied by the NPs against ClpP1P2, DprE1, InhA, KasA, PanK, PknB and Pks13, which were compared with the respective control inhibitor. This identified NPs which occupied the same “chemical space” as most of the anti-TB drugs. Only isoniazid, ethambutol, streptomycin, kanamycin, amikacin and levofloxacin, exhibited higher hydrophilicity compared to the NP. This could indicate that a few NPs have high cytotoxicity due to their higher relative lipophilicity. This will must be directly assessed through experimental testing.

Our structural study focused on bisbenzylisoquinoline alkaloids 2-nortiliacorinine, tiliacorine and 13′-bromotiliacorinine against the targets PknB and DprE1. These bisbenzylisoquinoline alkaloids isolated from *Tiliacora triandra* roots, which are used in Thai cuisine, were very effective in suppressing 59 isolated MDR-TB strains with MICs in the range of 1.5–6.25 µg·mL^−1^ [46]. Structurally, these molecules are similar, but the minor differences resulted in different binding properties. Tiliacorine, with the lowest binding energy, formed two hydrogen bonds with the residues Tyr94 and Phe19 of PknB. However, both nortiliacorinine and 13′-bromotiliacorine only formed one stable hydrogen bond with Gly97 and Tyr94 (Figure 2). The bromide substitution at C-13 of 13′-bromotiliacorine made the molecule less planar and thereby increased the binding energy through steric impedance as seen with the superimposed docked conformations of all three molecules (Figure 3). A key feature here revealed is the interactions of the hydrophobic core of these NPs and the planar dihydroxy anthraquinone moiety of the control in the hydrophobic “cage” of PnkB [18].

The importance of our modeling approach for drug optimization was demonstrated by considering the binding of DprE1 to tiliacorine, nortiliacorinine and 13′-bromotiliacorine. The interaction with these NPs is stabilized by several non-covalent interactions, but crucially, these were distinctive from the binding simulations with BTZ043, where H-bonding, hydrophobic and ionic interactions are responsible for the stabilization of the complex [61]. Additionally, the residue Cys387, before identified as critical for covalently binding to Ct325 (3-(hydroxyamino)-*N*-[(1R)-1-phenylethyl]-5-(trifluoromethyl) benzamide) is not involved in the binding of any of the NPs. Overall, 13′-bromo-tiliacorinine has shown slightly better anti-mycobacterial activity (and lower cytotoxicity against MRC-5 cell lines) than tiliacorine, nortiliacorinine, despite the higher binding energies here reported. Other biochemical assays are required to understand how the different chemical properties of these NPs influence bacterial uptake, metabolism and target binding. Nonetheless, the molecular interactions that we have defined can be used to inform chemical derivatization strategies aiming to increase specificity and decrease toxicity.

*α*-cubebin, a dibenzyl butyrolactone lignan, has been isolated from several species in various families, such as Aristolochiaceae, Myristicaceae, Rutaceae, and Piperaceae [62]. It is known to act as an insect antifeedant, as was noted with *Anticarsia gemmatalis* [63,64], as well as being anti-tubercular [24]. However, α-cubebin displays only moderate activity against several mono- and multi-drug-resistant isolates of *M. tuberculosis* (MICs ranging 50–100 µg·mL^−1^). Interestingly, it does not display cytotoxicity against LLCMK2 fibroblasts [65], suggesting that α-cubebin could merit derivatization to make it a better drug lead. α-cubebin exhibited a low binding energy value when docked to Pks13 and interacts with some of the key residues within Pks13 as the drug inhibitor I28. Additionally, unlike I28, α-cubebin has been predicted to bind to the protein tyrosine phosphatase B (PtpB) of *M. tuberculosis* [66]. This suggested that α-cubebin had some unique distinct binding characteristics with the *M. tuberculosis* proteome compared to 128. The information of *α*-cubebin’s binding site will facilitate the optimization of this compound towards greater efficacy and selectivity.

In conclusion, we show how four promising NPs—tiliacorine, nortiliacorinine, 13′-bromotiliacorine and α-cubebin—have very lower binding energies than the respective controls against three “druggable” anti-mycobacterial targets PnkB, DprE1 and Pks13. Due to problems in obtaining the NPs from natural sources or complex total synthesis, the predicted in silico activity/binding will greatly facilitate drug optimization prior to further studies. Even though in silico and in vitro results are not always correlated, our approach based on reverse docking will generate hypotheses that should inform further in vitro validation studies and aid the optimization of new and promising anti-TB natural products.

## 4. Materials and Methods

### 4.1. Selected Anti-Tubercular Natural Products

Information about the selected anti-mycobacterial NPs and their activity against TB in minimum inhibitory concentration (MIC) is given in Table 1.

### 4.2. Ligand and Protein Selection

A total of 53 NPs with reported anti-mycobacterial activity ≤100 mg·mL^−1^ were selected. All chemical structures were retrieved from the PubChem compound database (NCBI) (http://www.pubchem.ncbi.nlm.nih.gov). The crystal structures and respective controls of ClpP1P2 (PDB ID: 4U0G) [8], DprE1 (PDB ID: 6HEZ) [67], InhA (PDB ID: 1ENY) [14], KasA (PDB ID: 2WGE) [16], PanK type 1 (PDB ID: 4BFT) [68], PknB (PDB ID: 2FUM) [18] and Pks13 (PDB ID: 5V3X) [10] were retrieved from the RCSB Protein Data Bank (PDB) database (https://www.rcsb.org).

### 4.3. Physicochemical and Structural Properties

In silico prediction of physicochemical and structural properties of the NPs was performed using PaDEL-descriptor [69] including the descriptors: nHBAcc_Lipinski (acceptor H-bonds), nHBDon_Lipinski (donor H-bonds), nRotB (number of rotation bonds), TopoPSA (topological polar surface area), MW (molecular weight) and XLogP (prediction of logP based on the atom-type method). Chemical space analyses were conducted with the NPs and 14 anti-TB drugs (ethambutol, isoniazid, pyrazinamide, rifampicin, streptomycin, ciprofloxacin, levofloxacin, moxifloxacin, amikacin, kanamycin, linezolid, bedaquiline, clofazimine and delamanid), comparing the descriptors above. Unsupervised principal component analyses (PCA) were generated using the statistical analysis tool of Metaboanalyst 4.0 [70].

### 4.4. Docking

The extended PDB format, PDBQT, was used to coordinate files to include atomic partial charges [71]. All file conversions were performed using the open-source chemical toolbox Open Babel 2.3.2 [72]. The ligand and protein structures were optimized using AutoDock Tools software (AutoDock 1.5.6), which involved adding all hydrogen atoms to the macromolecule, which is a step necessary for correct calculation of partial atomic charges. Gasteiger charges are calculated for each atom of the macromolecule in AutoDock 1.5.6 [71].

NPs were docked against ClpP1P2, DprE1, InhA, KasA, PanK, *P*knB, and Pks13 along with each respective control inhibitors, ZIL (*N*-[(benzyloxy)carbonyl]-l-isoleucyl-l-leucine), BTZ043, isoniazid, TLM (thiolactomycin), ZVT, MIX, I28. Molecular docking calculations for all compounds with each of the proteins were performed using AutoDock Vina 1.1.2. Docking calculation was generated with the software free energy binding own scoring function. The binding affinity of the ligand was expressed in kcal·mol^−1^. Nine different poses were calculated for each protein with the parameters num_modes = 9 and exhaustiveness = 16. The lowest energy conformation was chosen for binding model analysis. Molecular interactions between ligand and protein were generated and analyzed by LigPlot^+^ and depicted by PyMOL. PyMOL Molecular Graphics System, Version 2.0 Schrödinger (http://www.pymol.org) was used to prepare Figures.

To provide enough space for free movements of the ligands, the grid box was constructed to cover the active sites as defined using AutoDock 1.5.6. The grid points for ClpP1P2 were set to 18 × 20 × 12, at a grid center of (x,y,z) −84.697, −2.336, 38.022 with a spacing of 1 Å. For DprE1, the grid points were set to 20 × 20 × 20, at a grid center of (x,y,z) 14.99, −20.507, 37.226 with a spacing of 1 Å. For InhA, the grid points were set to 26 × 24 × 22, at a grid center of (x,y,z) −5.111, 33.222, 13.410 with space ng of 1 Å. For KasA, the grid points were set to 20 × 20 × 20, at a grid center of (x,y,z) 38.342, −7.033, 13.410 with a spacing of 1 Å. For PanK, the grid points were set to 20 × 20 × 20, at a grid center of (x,y,z) −18.742, 13.919, 11.679 with a spacing of 1 Å. For PknB, the grid points were set to 21 × 20 × 20, at a grid center of (x,y,z) 61.518, 2.429, −25.588 with a spacing of 1 Å. For Pks13, the grid points were set to 16 × 18 × 14, at a grid center of (x,y,z) 3.954, 27.324, 8.499 with a spacing of 1 Å.

## Figures and Tables

**Table 1:** Plants and their molecules active against different *Mycobacterium* strains. ^a^ mono-resistant clinical and non-clinical isolates, ^b^ multidrug-resistant (MDR) clinical and non-clinical isolates, ^c^ mycobacteria other than *M. tuberculosis*, ^d^ *Mycobacterium tuberculosis.*

Plant Names	Active Phytomolecules	MIC (μg/mL)	References
*Andrographis paniculata*	Andrographolide	^-^	[19,20]
*Aristolochia brevipes* Benth.	6*α*-7-Dehydro-*N*-formyl-nornantenine	>50 ^a,b,d^	[21]
	*N*-Formylnornantenine	>50 ^a,b,d^	
	Aristolactam I	12.5–25 ^a,b,d^	
*Aristolochia taliscana* Hook and Arn.	Licarin A	3.12–25 ^a,b,d^	[22]
	Licarin B	12.5–5 ^a,b,d^	
	Eupomatenoid-7	6.25–50 ^a,b,d^	
*Aristolochia elegans* Mast.	Fargesin	12–50 ^a,b,d^	[23]
	(8R,8’R,9R)-Cubebin or *α*-Cubebin	50–100 ^a,b,d^	
*Artemisia capillaris* Thunb.	Ursolic acid	12.5–50 ^a,b,c,d^	[24,25]
	Hydroquinone	12.5–25 ^a,b,c,d^	[25]
*Azorella compacta* Phil., *A. madreporica* Clos.	Azorellanol	12.5 ^b,d^	[26]
	Mulin-11,13-dien-20-oic acid	25–50 ^b,d^	
	Mulinol	12.5–25 ^b,d^	
*Beilschmiedia tsangii* Merr.	Beilschmin A	2.5 ^d^	[27]
*Blepharodon nitidum* (Vell.) J.F. Macbr.	25-Hydroperoxycycloart-23-en-3*β*-ol	25 ^b^	[28]
*Citrullus colocynthis* (L.) Schrad.	Cucurbitacin-*E*-2-*o*-*β*-*d*-glucopyranoside	25–62.5 ^a,b,c,d^	[29]
*Clavija procera* B. Ståhl	Aegicerin	1.6–3.12 ^a,b,d^	[30]
*Curcuma longa* L.	Curcumin	100 ^d^	[31]
	Demethoxycurcumin	50 ^d^	
	Bisdemethoxycurcumin	25 ^d^	
*Diospyros anisandra* S.F. Blake	Plumbagin	1.5–62.5 ^b,c,d^	[32,33]
	Maritinone or 8,8′-Biplumbagin	3.12 ^b,d^	[32]
	3,3′-Biplumbagin	3.12 ^b,d^	
*Diospyros montana*	Diospyrin	8–250 ^b,c,d^	[33,34]
*Euclea natalensis* A. DC.	7-Methyljuglone	0.5–1.25 ^a,b,d^	[34]
	Mamegakinone	100 ^d^	[35]
	Isodiospyrin	10 ^d^	
	Neodiospyrin	10 ^d^	
	Shinanolone	100 ^d^	
*Ferula communis* Linn.	Ferulenol	1.25 ^c^	[36]
*Feniculum vulgare* Mill.	5-Hydroxy-furanocoumarin or bergaptol	100–200 ^b^	[37]
*Juniperus communis* subsp. communis var. communis L.	Totarol	2–25 ^a,c,d^	[38]
	Ferruginol	5 ^c^	[39]
	Sandaracopimeric acid	30 ^c^	
	4-Epiabietol	60 ^c^	
*Justicia adhatoda* L. or *Adhatoda vesica*	Vasicine	200 ^d^	
*Kaempferia galangal* L.	Ethyl-*p*-methoxycinnamate	50–100 ^b,d^	[40]
*Lantana hispida* Kunth	Oleanolic acid	25–100 ^a,b,c,d^	[24,41]
*Larrea tridentata* Coville	Dihydroguaiaretic acid	12–50 ^b,d^	[42]
	4-Epi-larreatricin	25–50 ^b,d^	
*Plectranthus grandidentatus* Gurke	Abietane	3.12–25 ^b,d^	[43]
*Plumeria bicolor* Ruiz and Pav.	Plumericin	1.5–2 ^b,d^	[44]
	Isoplumericin	2–2.5 ^b,d^	
*Struthanthus concinnus*	Obtusifoliol	50 ^d^	[45]
*Tabernemontana elegans* Stapf. or *Tiliacora triandra*	Tiliacorinine	3.12–6.25 ^b,d^	[46]
	2′-Nortiliacorinine	1.5–6.25 ^b,d^	
	13′-Bromotiliacorinine	1.5–6.25 ^b,d^	
*Ventilago madraspatana*	Emodin	4–128 ^b,c^	[33]
*Vetiveria zizanioides*	*α*-Curcumene	31.25–125 ^a,b,c^	[47]
	Valencene	62.5–250 ^a,b,c^	
	Selina-3,7(11)-diene		

## Data Availability

Data sharing not applicable. No new data were created or analyzed in this study.

## Supplementary Materials

The following are available online, Figure S1: Topological polar surface (TopoPSA) and binding energy of studied natural products against ClpP1P2, DprE1, InhA, KasA, PanK, PknB and Pks13, Figure S2: Molecular weight (MW) and binding energy of studied natural products against ClpP1P2, DprE1, InhA, KasA, PanK, PknB and Pks13, Figure S3: Partition coefficient (XLogP) and binding energy of studied natural products against ClpP1P2, DprE1, InhA, KasA, PanK, PknB and Pks13, Figure S4: Number of H-bonds acceptors (nHBAcc_Lipinski) and binding energy of studied natural products against ClpP1P2, DprE1, InhA, KasA, PanK, PknB and Pks13, Figure S5: Number of H-bonds donors (nHBDon_Lipinski) and binding energy of studied natural products against ClpP1P2, DprE1, InhA, KasA, PanK, PknB and Pks13, Figure S6: Number of rotational bonds (nRotB) and binding energy of studied natural products against ClpP1P2, DprE1, InhA, KasA, PanK, PknB and Pks13, Table S1: Structures of the selected natural products, Table S2: Structures of inhibitor (control) drugs for each mycobacterial target, Table S3: The physiochemical properties using PaDel-descriptor: molecular weight (MW), partition coefficient (xLogP), rotatable bonds (nRotB), H-bond donors (nHBDon_Lipinski), H-bond acceptors (nHBAcc_Lipinski) and topological polar surface area (TopoPSA), Table S4: List of ligand molecules chosen for testing with their corresponding drug likeness and medicinal chemistry filters using SWISS ADME.

## Sample Availability

Samples of the compounds are not available from the authors.
