# Supplementary material for: Molecular Docking Suggests the Targets of Anti-Mycobacterial Natural Products
Source: Molecules. 2021 Jan 18;26(2):475. doi: 10.3390/molecules26020475 (PMC7831053; doi:10.3390/molecules26020475)
Supplement: Supplementary file 1 [file molecules-26-00475-s001.pdf]

Supplementary Materials

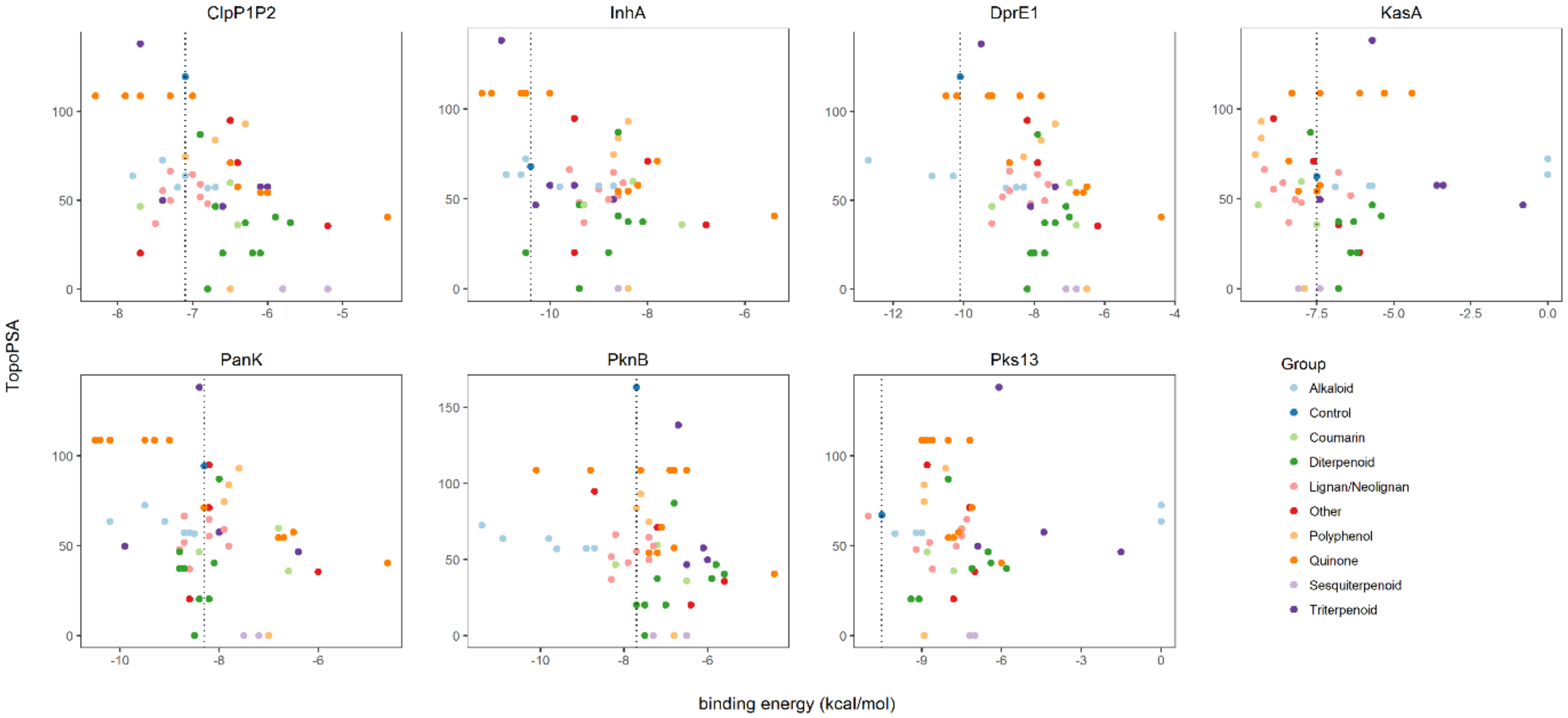

Supplementary Figure 1: Topological polar surface (TopoPSA) and binding energy of studied natural products against ClpP1P2, DprE1, InhA, KasA, PanK, PknB and Pks13.

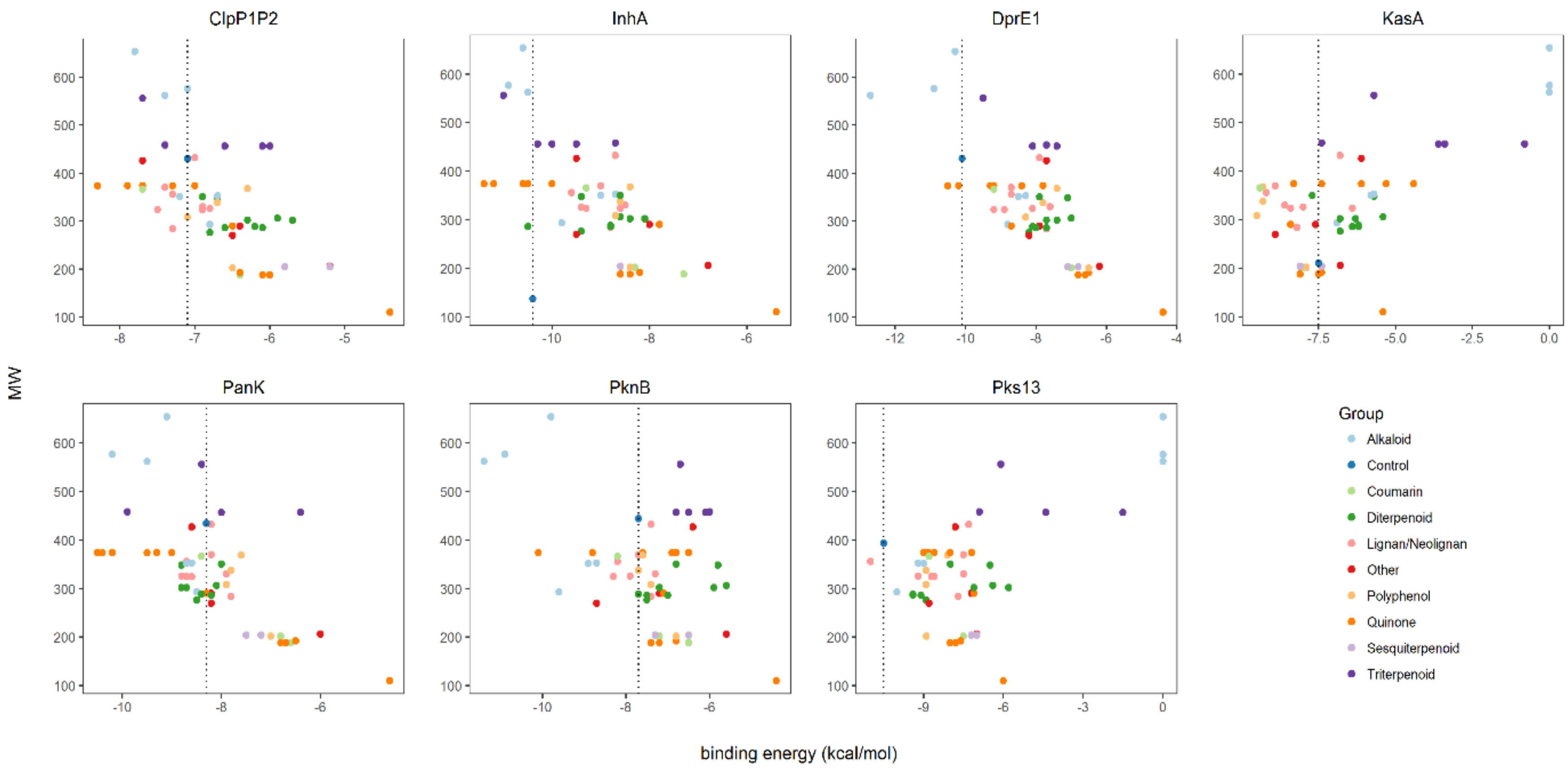

Supplementary Figure 2: Molecular weight (MW) and binding energy of studied natural products against ClpP1P2, DprE1, InhA, KasA, PanK, PknB and Pks13.

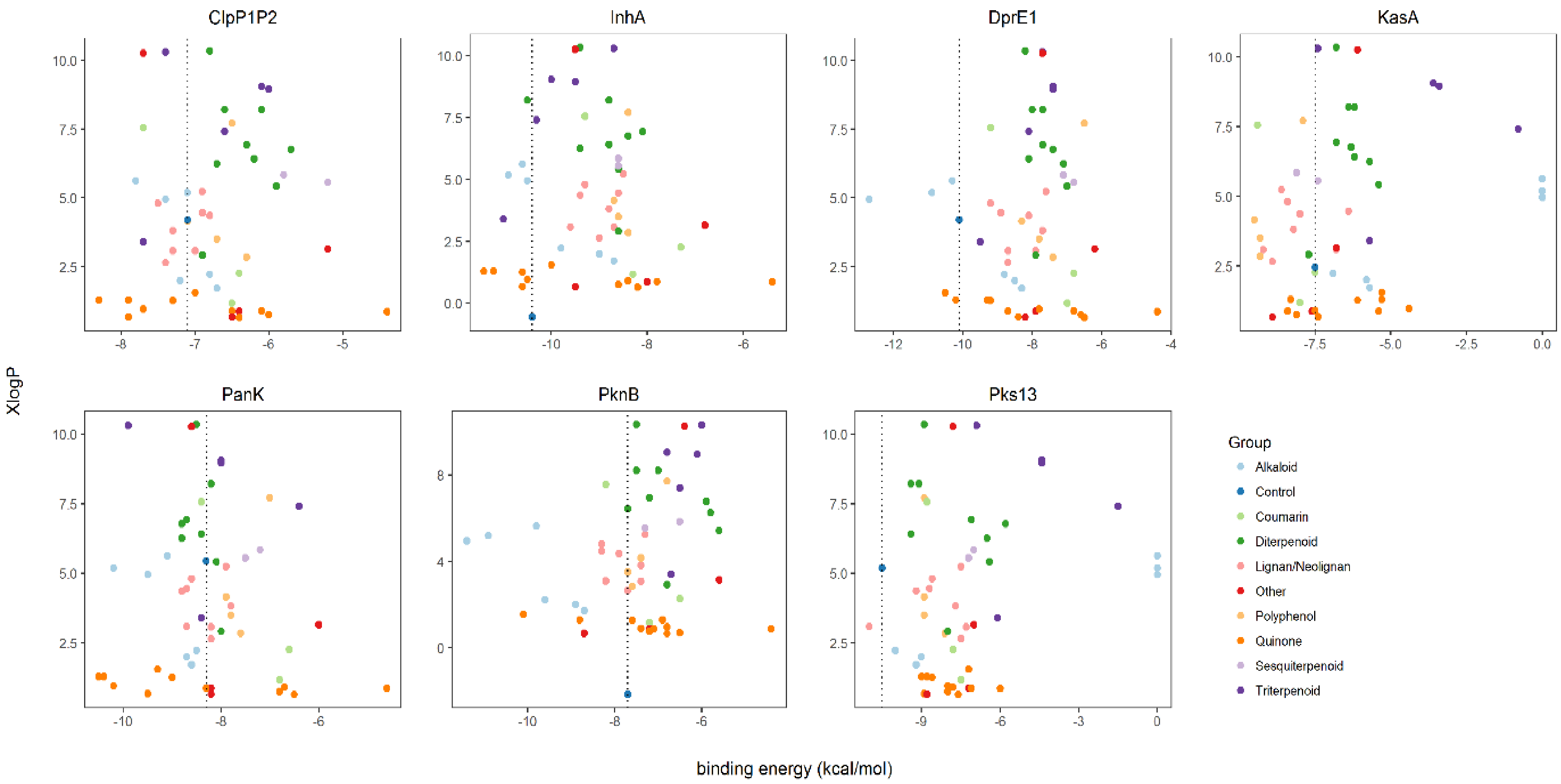

Supplementary Figure 3: Partition coefficient (XLogP) and binding energy of studied natural products against ClpP1P2, DprE1, InhA, KasA, PanK, PknB and Pks13.

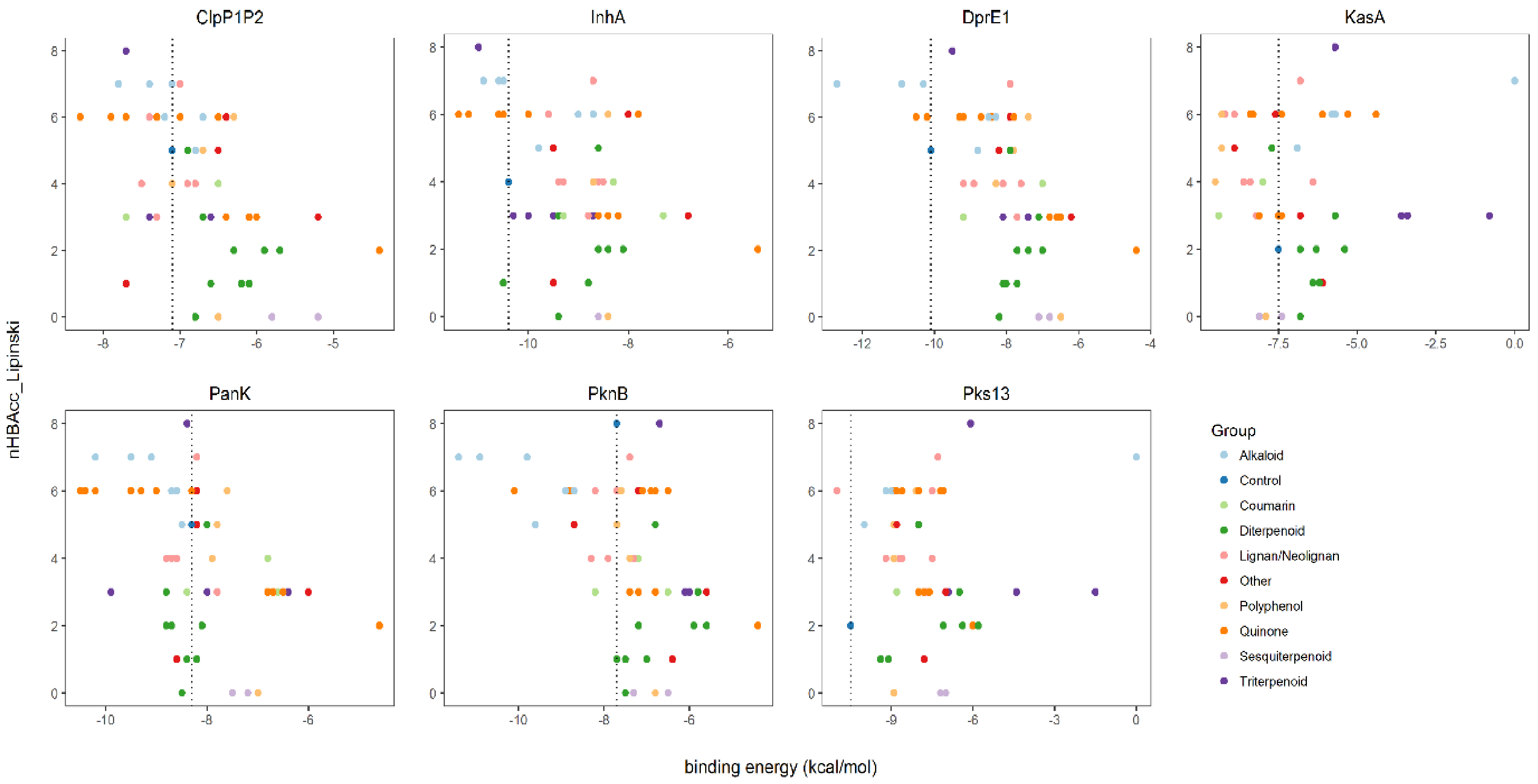

Supplementary Figure 4: Number of H-bonds acceptors (nHBAcc_Lipinski) and binding energy of studied natural products against ClpP1P2, DprE1, InhA, KasA, PanK, PknB and Pks13.

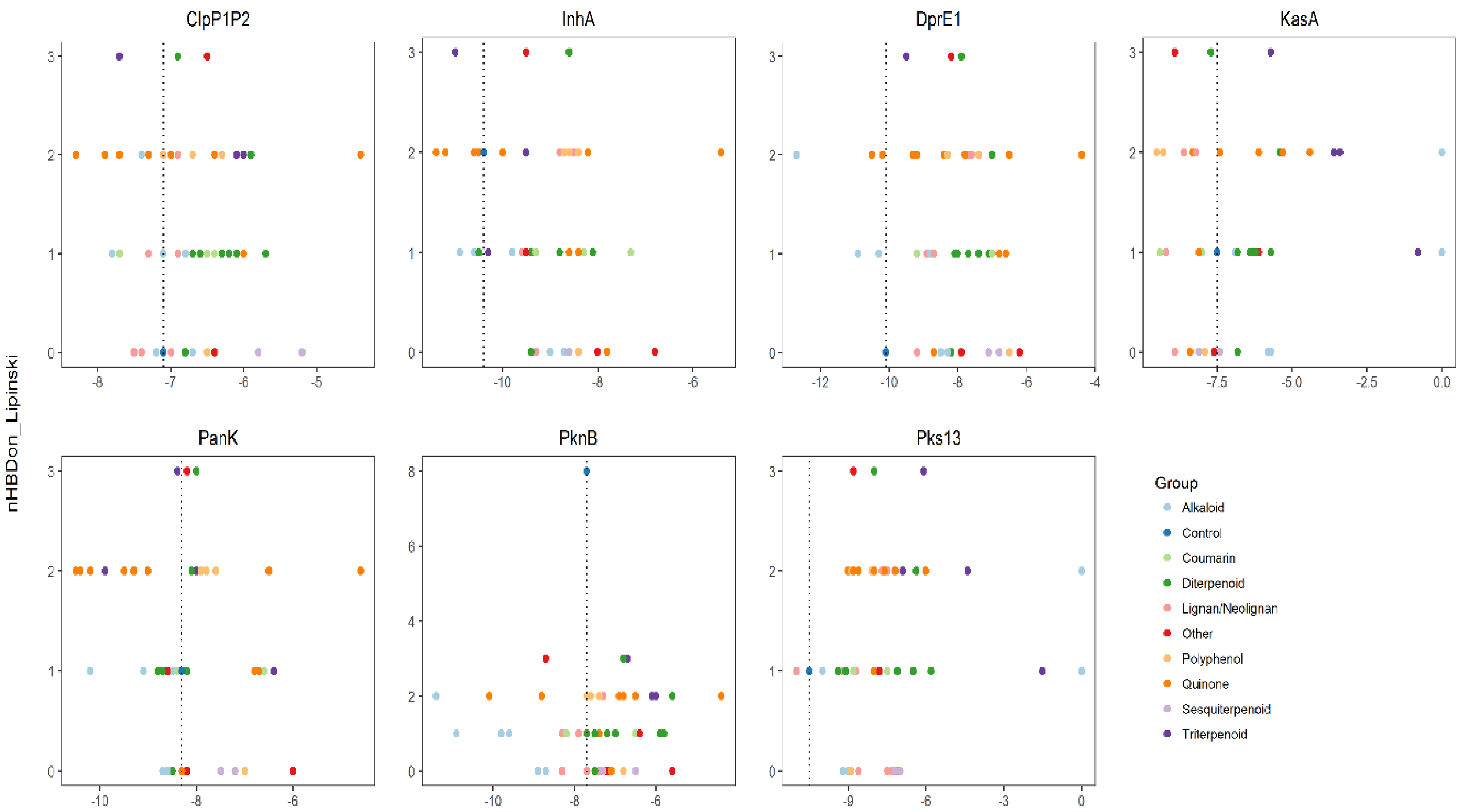

Supplementary Figure 5: Number of H-bonds donnors (nHBDon_Lipinski) and binding energy of studied natural products against ClpP1P2, DprE1, InhA, KasA, PanK, PknB and Pks13.

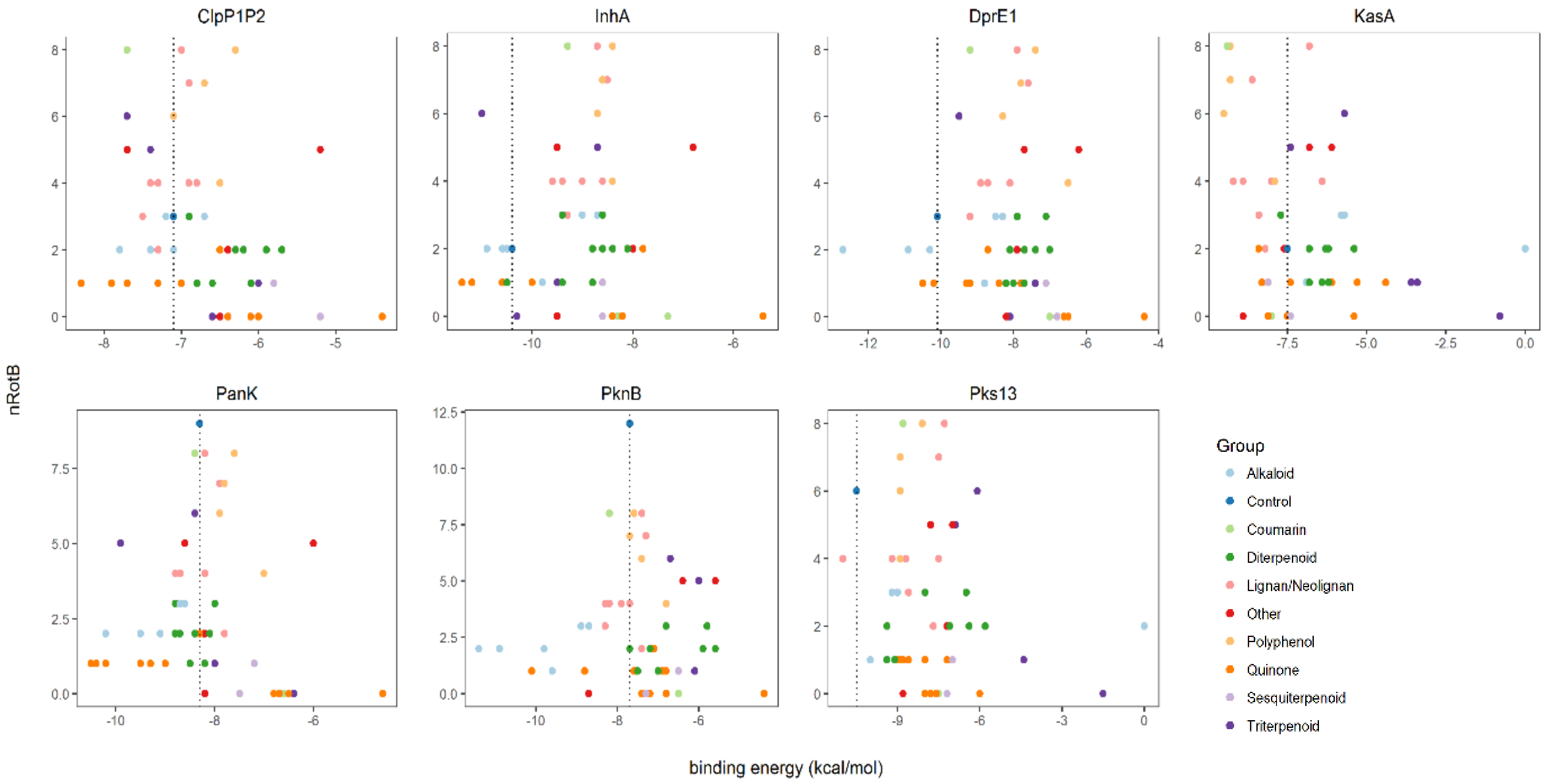

Supplementary Figure 6: Number of rotational bonds (nRotB) and binding energy of studied natural products against ClpP1P2, DprE1, InhA, KasA, PanK, PknB and Pks13.

Supplementary Table 1: The physiochemical properties using PaDel-Descriptor: molecular weight (MW), partition coefficient (xLogP), rotatable bonds (nRotB), H-bond donors (nHBDon_Lipinski), H-bond acceptors (nHBAcc_Lipinski) and topological polar surface area (TopoPSA)

	Group		nHBAcc_Lipinski	nHBDon_Lipinski	nRotB	TopoPSA	MW	XLogP	Pks13	PknB	PanK	KasA	InhA	DprE1	ClpP1P2
**Licarin A**	Lignan/Neolignan	1	4	1	4	47.92	326.151809	4.363	-9.2	-7.9	-8.8	-8	-9.4	-8.1	-6.8
**Licarin B**	Lignan/Neolignan	2	4	0	3	36.92	324.136159	4.809	-8.6	-8.3	-8.6	-8.4	-9.3	-9.2	-7.5
**eupomatenoid-7**	Lignan/Neolignan	3	4	1	4	51.83	324.136159	4.461	-8.7	-8.3	-8.7	-6.4	-8.6	-8.9	-6.9
**Aristolactam I**	Alkaloid	4	5	1	1	56.79	293.068808	2.229	-10	-9.6	-8.5	-6.9	-9.8	-8.8	-6.8
**Fargesin**	Neolignan	5	6	0	4	55.38	370.141638	2.649	-7.5	-7.7	-8.2	-8.9	-9	-8.7	-7.4
**alpha-Cubebin**	Lignan/Neolignan	6	6	1	4	66.38	356.125988	3.083	-11	-8.2	-8.7	-9.2	-9.6	-8.7	-7.3
**Ursolic acid**	Triterpenoid	7	3	2	1	57.53	456.360345	8.954	-4.4	-6.1	-8	-3.4	-9.5	-7.4	-6
**Hydroquinone**	Quinone	8	2	2	0	40.46	110.036779	0.87	-6	-4.4	-4.6	-5.4	-5.4	-4.4	-4.4
**azorellanol**	Diterpenoid	9	3	1	3	46.53	348.266445	6.251	-6.5	-5.8	-8.8	-5.7	-9.4	-7.1	-6.7
**Beilschmin A**	Lignan/Neolignan	10	7	0	8	64.61	432.214803	3.08	-7.3	-7.4	-8.2	-6.8	-8.7	-7.9	-7
**25-Hydroperoxycycloart-23-en-3beta-ol**	Triterpenoid	11	3	2	5	49.69	458.375996	10.306	-6.9	-6	-9.9	-7.4	-8.7	-7.7	-7.4
**cucurbitacin E**	Triterpenoid	12	8	3	6	138.2	556.303618	3.404	-6.1	-6.7	-8.4	-5.7	-11	-9.5	-7.7
**aegicerin**	Triterpenoid	13	3	1	0	46.53	456.360345	7.411	-1.5	-6.5	-6.4	-0.8	-10.3	-8.1	-6.6
**Diospyrin**	Quinone	14	6	2	1	108.74	374.079038	1.294	-9	-8.8	-10.4	-8.3	-11.2	-9.3	-8.3
**5-Hydroxy furanocoumarin or bergaptol**	Coumarin	15	4	1	0	59.67	202.026609	1.171	-7.5	-7.2	-6.8	-8	-8.3	-7	-6.5
**vasicine**	Coumarin	16	3	1	0	35.83	188.094963	2.268	-7.8	-6.5	-6.6	-7.5	-7.3	-6.8	-6.4
**Ethyl 4-Methoxycinnamate**	Other	17	3	0	5	35.53	206.094294	3.149	-7	-5.6	-6	-6.8	-6.8	-6.2	-5.2
**Oleanolic acid**	Triterpenoid	18	3	2	1	57.53	456.360345	9.052	-4.4	-6.8	-8	-3.6	-10	-7.4	-6.1
**Dihydroguaiaretic acid**	Lignan/Neolignan	19	4	2	7	58.92	330.183109	5.238	-7.5	-7.3	-7.9	-8.6	-8.5	-7.6	-6.9
**4-Epi-larreatricin**	Lignan/Neolignan	20	3	2	2	49.69	284.141245	3.818	-7.7	-7.4	-7.8	-8.2	-8.8	-7.7	-7.3
**Abietane**	Diterpenoid	21	0	0	1	0	276.281701	10.347	-8.9	-7.5	-8.5	-6.8	-9.4	-8.2	-6.8
**Plumericin**	Other	22	6	0	2	71.06	290.079038	0.877	-7.2	-7.2	-8.2	-7.6	-8	-7.9	-6.4
**Tiliacorinine**	Alkaloid	24	7	2	2	72.42	562.246772	4.952	3.7	-11.4	-9.5	32.7	-10.5	-12.7	-7.4
**2'-Nortiliacorinine**	Alkaloid	25	7	1	2	63.63	576.262422	5.191	1.4	-10.9	-10.2	28.3	-10.9	-10.9	-7.1
**Plumbagin**	Quinone	26	3	1	0	54.37	188.047344	0.756	-8	-7.2	-6.8	-8.1	-8.6	-6.6	-6
**Maritinone or 8,8í-biplumbagin**	Quinone	27	6	2	1	108.74	374.079038	0.68	-8.9	-6.5	-9.5	-7.4	-10.6	-8.4	-7.9
**3,3í-biplumbagin**	Quinone	28	6	2	1	108.74	374.079038	1.266	-8.6	-7.6	-9	-6.1	-10.6	-9.2	-7.3
**6?-7-dehydro-N formyl-nornantenine**	Alkaloid	29	6	0	3	57.23	351.110673	2	-9	-8.9	-8.7	-5.8	-9	-8.5	-7.2
**N-formylnornantenine**	Alkaloid	30	6	0	3	57.23	353.126323	1.717	-9.2	-8.7	-8.6	-5.7	-8.7	-8.3	-6.7
**Mulin-11,13-dien-20-oic acid**	Diterpenoid	31	2	1	2	37.3	302.22458	6.773	-5.8	-5.9	-8.8	-6.3	-8.4	-7.4	-5.7
**Mulinol**	Diterpenoid	32	2	2	2	40.46	306.25588	5.423	-6.4	-5.6	-8.1	-5.4	-8.6	-7	-5.9
**Curcumin**	Polyphenol	35	6	2	8	93.06	368.125988	2.847	-8.1	-7.6	-7.6	-9.3	-8.4	-7.4	-6.3
**demethoxycurcumin**	Polyphenol	36	5	2	7	83.83	338.115424	3.502	-8.9	-7.7	-7.8	-9.3	-8.6	-7.8	-6.7
**bisdemethoxycurcumin**	Polyphenol	37	4	2	6	74.6	308.104859	4.157	-8.9	-7.4	-7.9	-9.5	-8.7	-8.3	-7.1
**Isodiospyrin**	Quinone	38	6	2	1	108.74	374.079038	0.964	-8	-6.8	-10.2	-4.4	-10.5	-7.8	-7.7
**Mamegakinone**	Quinone	39	6	2	1	108.74	374.079038	1.55	-7.2	-10.1	-9.3	-5.3	-10	-10.5	-7

**7-methyljuglone**	Quinone	40	3	1	0	54.37	188.047344	0.898	-7.8	-7.4	-6.7	-7.5	-8.4	-6.8	-6.1
**Neodiospyrin**	Quinone	41	6	2	1	108.74	374.079038	1.294	-8.8	-6.9	-10.5	-5.3	-11.4	-10.2	-7.9
**Shinanolone**	Quinone	42	3	2	0	57.53	192.078644	0.653	-7.6	-6.8	-6.5	-7.4	-8.2	-6.5	-6.4
**isoplumericin**	Quinone	46	6	0	2	71.06	290.079038	0.877	-7.1	-7.1	-8.3	-8.4	-7.8	-8.7	-6.5
**13?-bromo-tiliacorinine**	Alkaloid	47	7	1	2	63.63	654.172934	5.625	4.6	-9.8	-9.1	52.3	-10.6	-10.3	-7.8
**a-curcumene**	Polyphenol	49	0	0	4	0	202.172151	7.712	-8.9	-6.8	-7	-7.9	-8.4	-6.5	-6.5
**valencene**	Sesquiterpenoid	50	0	0	1	0	204.187801	5.846	-7	-6.5	-7.2	-8.1	-8.6	-7.1	-5.8
**Selina-3,7(11)-diene**	Sesquiterpenoid	51	0	0	0	0	204.187801	5.553	-7.2	-7.3	-7.5	-7.4	-8.6	-6.8	-5.2
**Emodin**	Other	52	5	3	0	94.83	270.052823	0.664	-8.8	-8.7	-8.2	-8.9	-9.5	-8.2	-6.5
**Andrographolide**	Diterpenoid	53	5	3	3	86.99	350.209324	2.913	-8	-6.8	-8	-7.7	-8.6	-7.9	-6.9
**Obtusifoliol**	Other	54	1	1	5	20.23	426.386166	10.259	-7.8	-6.4	-8.6	-6.1	-9.5	-7.7	-7.7
**Totarol**	Diterpenoid	55	1	1	1	20.23	286.229666	8.211	-9.4	-7	-8.2	-6.2	-10.5	-7.7	-6.1
**Ferruginol**	Diterpenoid	56	1	1	1	20.23	286.229666	8.211	-9.1	-7.5	-8.2	-6.4	-8.8	-8	-6.6
**sandaracopimeric acid**	Diterpenoid	57	2	1	2	37.3	302.22458	6.935	-7.1	-7.2	-8.7	-6.8	-8.1	-7.7	-6.3
**4-Epiabietol**	Diterpenoid	58	1	1	2	20.23	288.245316	6.42	-9.4	-7.7	-8.4	-6.2	-8.8	-8.1	-6.2
**ferulenol**	Coumarin	59	3	1	8	46.53	366.219495	7.559	-8.8	-8.2	-8.4	-9.4	-9.3	-9.2	-7.7
	Control	ZIL	7	3	13	104.73	378.215472	5.054							-7.1
	Control	BTZ043	5	0	3	119.57	431.076276	4.203						-10.1	
	Control	I28	2	1	6	66.94	393.194008	5.19	-10.5						
	Control	INH	4	2	2	68.01	137.058912	-0.571					-10.4		
	Control	MIX	8	8	12	163.18	444.200885	-2.166		-7.7					
	Control	TLM	2	1	2	62.6	210.071451	2.458				-7.5			
	Control	ZVT	5	1	9	94.34	434.097953	5.441			-8.3				
